# Video-based feedback using real consultations for a formative assessment in communication skills

**DOI:** 10.1186/s12909-020-1955-6

**Published:** 2020-02-24

**Authors:** M. C. Dohms, C. F. Collares, I. C. Tibério

**Affiliations:** 1 Center for Development in Medical Education, University of Sao Paulo, Av Dr. Arnaldo, São Paulo, 01246-903 Brazil; 2 Department of Educational Development and Research, School of Health Professions, Education, Maastricht University, PO Box 616, 6200MD Maastricht, The Netherlands

**Keywords:** Communication skills, Physician/patient relationship, Primary care education, Testing/assessment, Postgraduate training

## Abstract

**Background:**

Pre-recorded videotapes have become the standard approach when teaching clinical communication skills (CCS). Furthermore, video-based feedback (VF) has proven to be beneficial in formative assessments. However, VF in CCS with the use of pre-recorded videos from real-life settings is less commonly studied than the use of simulated patients.

To explore: 1) perceptions about the potential benefits and challenges in this kind of VF; 2) differences in the CCC scores in first-year medical residents in primary care, before and after a communication program using VF in a curricular formative assessment.

**Method:**

We conducted a pre/post study with a control group. The intervention consisted of VF sessions regarding CCS, performed in a small group with peers and a facilitator. They reviewed clinical consultations pre-recorded in a primary care setting with real patients. Before and after the intervention, 54 medical residents performed two clinical examinations with simulated patients (SP), answered quantitative scales (Perception of Patient-Centeredness and Jefferson Empathy Scale), and semi-structured qualitative questionnaires. The performances were scored by SP (Perception of Patient-Centeredness and CARE scale) and by two blind raters (SPIKES protocol-based and CCOG-based scale). The quantitative data analysis employed repeated-measures ANOVA. The qualitative analysis used the Braun and Clarke framework for thematic analysis.

**Results:**

The quantitative analyses did not reveal any significant differences in the sum scores of the questionnaires, except for the Jefferson Empathy Scale. In the qualitative questionnaires, the main potential benefits that emerged from the thematic analysis of the VF method were self-perception, peer-feedback, patient-centered approach, and incorporation of reflective practices. A challenging aspect that emerged from facilitators was the struggle to relate the VF with theoretical references and the resident’s initial stress to record and watch oneself on video.

**Conclusion:**

VF taken from real-life settings seems to be associated with a significant increase in self-perceived empathy. The study of other quantitative outcomes related to this VF intervention needs larger sample sizes. VF with clinical patients from real healthcare settings appears to be an opportunity for a deeper level of self-assessment, peer-feedback, and reflective practices.

## Background

Currently, pre-recorded videotapes have become the standard approach when teaching clinical communication skills (CCS) [1]. Video feedback (VF) has shown positive effects in skills training and formative assessment [2, 3]. However, what do we know about video-based feedback in CCS using pre-recorded videos from real-life settings? Although the closer the assessment is to reality, the more valid it is likely to be [4], studies about VF using pre-recorded videos in a real-life setting have been less frequent than video feedback with simulated patients. Studies regarding the effects of different VF methodologies using real medical consultations are still scarce [5].

Feedback based on consultations recorded on video has many advantages over feedback performed directly after the observation. The video format allows for multiple reviews of the consultation as well as a more careful analysis of nonverbal communication. Consequently, VF may facilitate reflection, self-assessment, and more active engagement of the participants in solving the observed problems [6–8]. Using real consultations allows for a real-life setting analysis and, thus, a better formative assessment [4, 5]. VF allows a better exploration of misunderstandings, disagreement factors as well as to investigate the patient’s responsiveness to specific doctor behaviors [9]. Video is the only method that enables learners to reflectively “look at themselves from a distance”, as a realistic painting of their skills [2, 10]. While it may seem threatening to learners at first, it can be potentially more stimulating and rewarding [1, 2].

Video review in small groups with a facilitator and peer feedback is more beneficial than the traditional feedback on the students’ communication skills as it enables a more detailed analysis of the learner’s behavior [11–13]. Moreover, the self-reflection process during the video review seems to be a practical approach to learning communication and professional behaviors [5, 14]. Besides, feedback becomes more useful for optimizing performance when combined with self-assessment, external feedback, and peer-feedback [15].

The purpose of this study is to explore: 1) perceptions about potential benefits and challenges in VF; 2) differences in the CCC scores in first-year medical residents in primary care, before and after a communication program using VF in a curricular formative assessment. The VF methodology used video pre-recorded in real-life settings, problem-based interviewing (PBI), and agenda-led outcome-based analysis (ALOBA) feedback in small groups with peers.

## Methods

### Design, setting and participants

We conducted a pre/post study with a control group to evaluate how an educational program grounded on video-based feedback influenced the medical residents’ communication skills. A group with VF sessions represented the intervention. All the residents in the group belonged to the same residency program. The educational intervention VF and the activity with simulated patients were curricular activities in the program, and the entire group was invited to participate in this study. The control group was similar to the intervention group. Everyone in both groups had the same supervisors and the same theoretical classes. The only difference was in a few local supervisors in both groups.

All first-year medical residents (N: 61) in an integrated primary care program in Brazil were invited to participate, and 54 completed all phases of the study (17 male and 37 female). The residents were randomly divided into small groups of 12 to 15 participants for the communication program with video feedback. We used the simulated patients to evaluate differences in the performance of the medical residents before and after the educational intervention: video feedback of consultations pre-recorded in a real-life setting.

All medical residents performed two simulated patient (SP) consultations in a video-recorded clinical performance examination before and two after VF sessions. The SPs were trained to role-play two clinical consultations in primary care: breaking bad news (HIV result and gastric cancer result) and a common clinical situation (migraine, hypertension, and back pain) for 7 min each. Two blind raters assessed the videotapes. They scored performance items related to communication skills in the 224 (4 videos by resident). Additionally, participants answered quantitative questionnaires (about the perception of patient-centeredness and empathy) before and after the intervention.

**Fig. 1:**
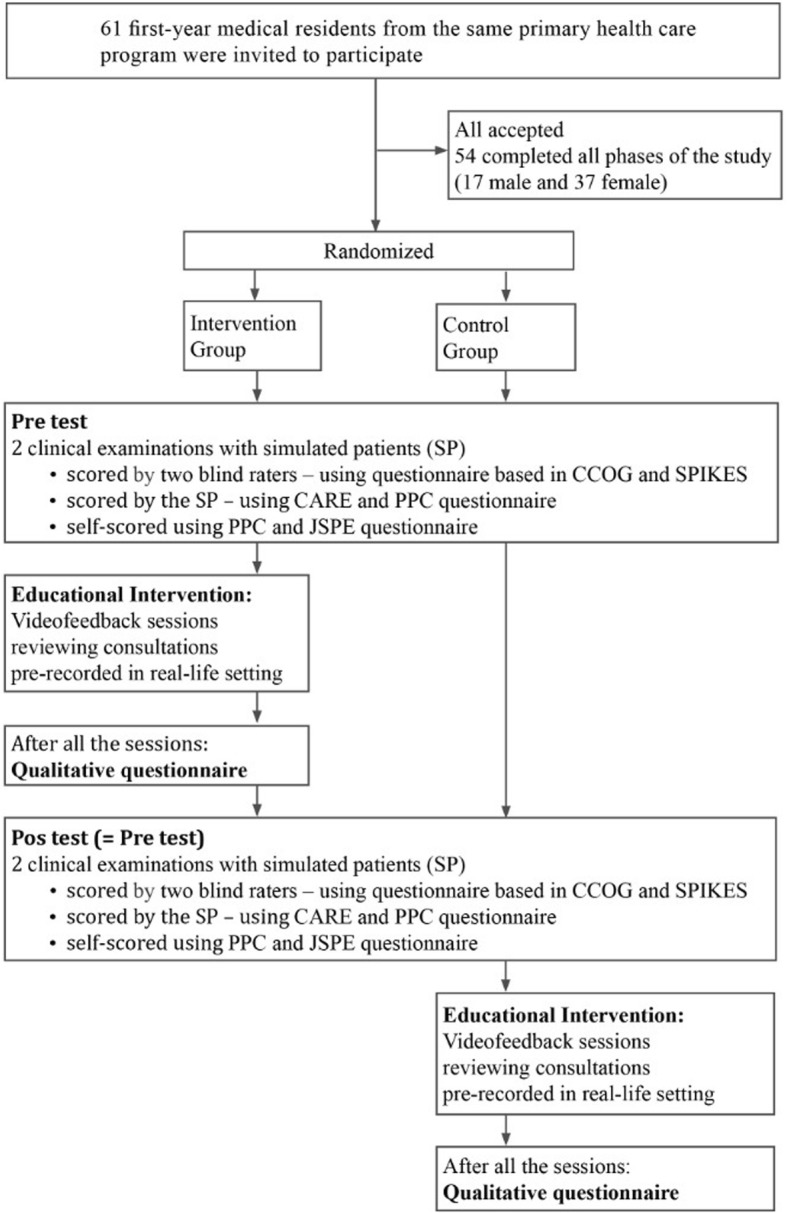
Diagram resuming the design of the study

The control group also experienced the intervention VF sessions after all the assessments were completed to avoid any potential educational disadvantage from not having the intervention. At the end of the sessions, both groups answered qualitative questions about their perceptions of the method. Therefore the control group was also able to answer the qualitative questionnaire (Fig. 1).

### The intervention

The VF methodology was based on Lesser’s PBI model [16] and an agenda-led outcome-based analysis (ALOBA) feedback [17, 18]. Each medical resident presented a pre-recorded interview in a real-life setting to a group of peers, subsequently receiving feedback from colleagues and two facilitators. The participants, including facilitators, did not change, and the facilitators were the same in both groups.

The communication program with VF commonly lasted about 10 weeks, with each video feedback session lasting around 90 min. There was no limit to the length of the recorded video, and each video-recorded media was 20 min long. In order to facilitate self-assessment and reflective practices, all videos were taped as close as possible to the session. The participants did not record any physical exam on tape.

In the VF session, each medical resident presented a video of a real consultation with some difficulty in medical communication. The facilitators and resident interviewer then agreed on an agenda addressing the topics in the video session [18]. The facilitators coordinated the VF session and facilitated the process of perceiving and understanding their self-image, performing a self-assessment, and finding new strategies by themselves. In this discussion, previous professional experiences from other participants, including the supervisor, illustrated different ways to improve upon the encountered difficulties [19–21].

The group watched the consultation as if they were conducting the consultation themselves, often pausing the video when someone addressed an issue. When the video stopped, the resident interviewer was invited to verbalize their self-image and what they observed in the interaction as well as analyze communication micro-skills and perform a microanalysis of micro-behaviors, paying close attention to the exact words spoken as well as non-verbal communication [11, 22]. The group then assisted the interviewer to find alternatives to the less effective behaviors identified [23]. We could argue that the inputs from the supervisor and the group also played a role in reinforcing positive behavior.

### Assessment instruments

We used seven instruments designed to assess the effects on communication skills, in checklist format with a Likert scale, completed after each clinical performance examination, before and after the intervention:

Questionnaires completed by the standardized SP:

1. Consultation and Relational Empathy (CARE) [24, 25]
2. Perception of Patient-Centeredness (PPC) [26]

Questionnaires completed by the medical residents:

3. Jefferson Scale of Physician Empathy (JSPE) [27, 28]
4. Perception of Patient-Centeredness (PPC) [26]
5. Qualitative questionnaire created by the authors, with three questions:
  What are your perceptions about the VF sessions?
  Were there any changes in your clinical practice after you began attending VF sessions? If so, please specify.
  Exemplify case situations presented and discussed during the sessions that led to changes in your daily practice.

Questionnaires completed by raters randomly watching videos of the clinical skills practical exam:

6. Questionnaire-based on Calgary-Cambridge Observation Guide (CCOG) [1, 18], with 17 items.
7. Questionnaire-based on SPIKES protocol [29], with 15 items

### Data analysis

The sum scores of the questionnaires pre- and post-interventions were analyzed in the control and intervention groups using mixed-design ANOVA. The qualitative data analysis used the Braun and Clarke framework for thematic analysis [30]. The themes were constructed from the reviewed data rather than from a preconceived theoretical stance. For the thematic analysis, the authors have read and double-checked the sentences and coded them. Researchers categorized recurring ideas into themes and sub-themes.

## Results

### Quantitative results

The following table summarizes the main results of the repeated measures ANOVA for between and within measures effects for the sum scores of the quantitative instruments used in the study (Table 1).

**Table 1:** Main results of the repeated measures ANOVA for between- and within-measures effects for the sum scores of the quantitative instruments used in the study.

		F	Sig (*p*)	Partial eta squared (η2)
CARE	Intercept	2016.999	0.000	0.988
	Group	0.363	0.553	0.015
	Time	0.824	0.373	0.033
	Time*Group	1.122	0.300	0.045
Jefferson	Intercept	14926.220	0.000	0.997
	Group	0.052	0.821	0.001
	Time	0.952	0.335	0.022
	Time*Group	5.494	0.024	0.113
PPC (answered by a simulated patient)	Intercept	1421.792	0.000	0.976
	Group	1.105	0.300	0.031
	Time	12.138	0.001	0.258
	Time*Group	3.558	0.068	0.092
PPC (answered by a medical resident)	Intercept	2348.669	0.000	0.982
	Group	1.152	0.289	0.026
	Time	72.210	0.000	0.621
	Time*Group	0.667	0.418	0.015
CCOG-based	Intercept	1667.467	0.000	0.951
	Group	2.075	0.153	0.024
	Time	3.231	0.076	0.036
	Time*Group	0.935	0.336	0.011
SPIKES protocol-based	Intercept	884.141	0.000	0.916
	Group	0.017	0.898	0.000
	Time	17.274	0.000	0.176
	Time*Group	0.096	0.758	0.001

The quantitative results did not reveal significant differences in most questionnaires:

- **CARE** [24, 25]**:** One item (*How do you evaluate the doctor’s performance in making a plan of action?*) presented a significant difference within applications, with a moderate effect size. The remaining items did not show any significant differences. As for the total scores, none of the results of the mixed ANOVA was significant, with small effect sizes for the within, between and interaction effects.
- **PPC** [26]**:** One item (*Regarding today’s problem, to what extent did you discuss personal issues with the doctor that might be affecting your health?*) had a significant difference within assessments. The remaining items did not show any significant differences. In the comparison between answers by SPs and residents, the medical residents had given themselves significantly lower grades, with a large effect size. When we separately analyzed the SPs’ and residents’ data, the differences that could be attributed to the intervention were not significant. The interaction between time and group had a small effect size only when data from SPs and residents were taken into account concurrently.
- **Jefferson Scale of Empathy (JSPE)** [27, 28]**:** none of the results regarding differences between groups before and after the intervention in terms of individual items were significant, with effect sizes close to null. For the total scores, the control group had lower mean total scores in the second assessment (from 82,33 to 80,94), and the intervention group had higher mean total scores after the intervention (from 80,26 to 83,63). As a result, a significant interaction with moderate effect size arose between group and time of application.
- **Checklist based on SPIKES protocol** [29]: only one item had significant effects within assessments (*Warning the patient that bad news is coming*), and the effect size was small. For total scores, there was a significant increase in scores with moderate effect size. This difference was not significant between the control and intervention groups and had a null effect.
  The **Checklist based on CCOG** [1, 18] showed no significant differences whatsoever.

### Qualitative results

The following table shows the main themes and sub-themes with supporting citations during the qualitative analysis (Table 2).

**Table 2:** Generated themes and sub-themes with supporting quotations from perceptions about the intervention

General perceptions about the program	“It was great! The program could have been further expanded (2.10)” “Distressing!” (3.1) “...very important to our learning (1.6)” “Innovative and motivating”(2.1) “Transforming (3.4)”	
Review of the video pre-recorded in a real-life setting		
Self-perceptions about CCS	Reviewing the own video	“It is hard to have a self-perception of our consultation, and the video feedback allows us to see our limitations and strengths.” (1.1).
		“I realized that I was authoritarian (3.16)”
	Reviewing the colleague’s video	“We learned with all the videos. We could see traits and actions in colleagues that also apply to us, correcting our postures and attitudes (2.11)”
Uncomfortable with the video	“I don’t like to see myself on video (1.5)” “The idea of recording myself in video and showing it to the group was stressful at first, but after I relaxed.” (2.9)	
Improved learning with challenging consultations	“I learned more with the more difficult consultations, like breaking bad news, when a patient demands an unnecessary exam and patients who talk too much (2.6)”	
Group feedback		
Learning new communication strategies from peer feedback	“I learned with my colleagues how to talk less and to interrupt less (3.7)”	
Better experience in receiving and providing feedback	“We learned to provide feedback and to put ourselves in the shoes of the interviewer (2.8)”	
Importance of feedback associated with theoretical references by the facilitator	“It could have been better integrated with the theory (3.4)”	
Differences perceived in medical practice after the intervention		
Better patient-centered approach	“I learned to allow the patient to talk more and to direct my focus on the patient’s problem (1.5)” “I improved in sharing my decision with the patient (3.16)”	
Awareness about non-verbal communication	“I began to better perceive non-verbal communication in patients and myself” (2.10)	
Changes in emotions reactions	“I felt more self-confident during the consultations (1.6)” “I disarmed myself!” (3.13) “I became calmer and more attentive (1.8).”	
Better consultation organization	“I improved the organization during my consultations” (2.14) “I improved the organization of my records (3.17).”	
Incorporation of self-reflexivity	“I started to further reflect about my practices (2.18)” “I started to pay further attention to my difficulties during the consultations (1.3)”	

All of the residents considered the educational intervention helpful for improving their communication skills. Some of them realized that the VF sessions were the only moment in their educational training in which they could look at themselves and observe from an outside perspective. Some residents found the experience motivational and helpful for more challenging consultations. Some of the situations described were: breaking bad news, leading with a verbose patient or with multiple demands, and denying the patient’s requests.

The primary potential benefits identified in the VF sessions were the possibility to self-perceive their communicative limitations while reviewing their videos as well as their peers’ videos. The residents stated that they were able to observe communication aspects in need of improvement and to make changes in their medical practice with more reflective practice.

“*I realized in my video that I was authoritarian and now I think I am better in sharing decisions with the patients* (male, 3.16)”; “*I started to pay further attention to my difficulties during the consultations* (female, 1.3)”

Other positive perceptions were related to the peer-feedback on communication skills. Many participants observed that they learned new communication strategies from their colleagues’ feedback for a better patient-centered approach. Furthermore, participants described the experience of providing feedback in the group as useful to the improvement of feedback skills. The residents also mentioned having further control over their emotional reactions and feeling more self-confident and calmer in interactions with patients after the VF sessions. They also reported improvements when organizing a consultation.

Some challenges related to the intervention: two residents reported that the experience of being videotaped and later watching themselves with the group was uncomfortable. However even so, they enjoyed the group discussion and watching their colleagues’ videos.

*“I don’t like to see myself on video* (male, 1.5); *The idea of recording myself in video and showing it to the group was stressful at first, but after I relaxed* (female 2.9).”

Some residents suggested more VF sessions and further correlations with theoretical references.

## Discussion

The results suggest that the intervention had a positive effect on self-reported levels of empathy on the Jefferson scale. The influence of preceptors and other residents during the supervision in primary care might have played a role. Therefore, we cannot assign the observed differences exclusively to the VF intervention. Perhaps the intervention was not sufficiently long and intense to produce measurable differences. Furthermore, the scholarly literature lacks a precise quantification as to the effects of VF since most studies have used narrative reviews [2].

The small sample size, a limitation given by the study setting, has likely caused this study to suffer from an underpowered analysis. Therefore, the small to moderate effect sizes might not emerge as significant in the mixed-design ANOVA. Other factors influencing the paucity of quantitative results are cognitive biases known to occur on raters’ behavior, such as the halo and the ceiling effects.

Some of the residents’ self-reported perceptions of the actual changes in their clinical practice seem hard to verify, particularly when the changes are related to professional attitudes and non-verbal communication. Besides, professionalism varies according to language and cultural context [31, 32]. In a meta-analysis, we found more statistical differences related to the influence of video feedback in verbal behavior than non-verbal behavior, more in reception skills than relation skills, and more in molar-skills than micro-skills [2]. Moreover, it is advisable to associate narratives and global ratings to checklists as well as an effective standardization of evaluators. Evidence demonstrates that on OSCE-type assessments, reliability seems to depend more on assessors than on objectivity [33–35].

As for the qualitative evaluation, our study confirmed that the intervention is a well-accepted method for a formative evaluation of communication skills [2, 36]. The video feedback recorded in a real-life setting allowed residents to revisit particular points in the real interview and gain a deeper understanding of a specific phrasing or behavior. Some residents also reported improvements in their self-confidence as well as behavioral changes, as seen in other studies [13, 37].

The research findings signpost some essential elements to consider when preparing a video feedback session to potentiate learning as well as a better understanding of the objectives, advantages, and challenges. The participants confirmed that emphasis on self-assessment and peer-feedback are positive dimensions of a formative assessment in a communication program [38]. When learners receive thoughtful comments by peers in a timely and confidential manner, supported by reflections, they find the process compelling, insightful, and instructive [39]. Moreover, as reported by the participants, when judging the work of others, learners can gain insights into their performances [40].

The participants also agreed that this VF methodology has the potential to improve the students’ feedback skills and provide a better acceptance of receiving feedbacks [41, 42]. Providing high-quality feedback is a challenge; furthermore, this is an essential skill for developing collaborative behavior when working in teams. Peer-feedback from colleagues is an important element of multi-source feedback, which is key to programmatic assessments, and reflective practice is an essential skill for effective learning [43].

On the other hand, residents reported critical challenges for facilitator skills, particularly the need to quickly establish connections between the given feedback with pertinent theoretical frameworks and the discomfort of watching themselves with the group. This experience was perceived as a stressful and unpleasant event in other studies [5, 35]. However, evidence suggests that the first video recording experience tends to be more stressful as the learners’ stress gradually decreases over time [35].

Equally significant is how the role of the facilitator is essential for preserving a pleasant and trustful atmosphere in VF [17, 22]. It is, therefore, crucial to have a mindful facilitator, attentive to the students’ psychological needs, and able to associate the feedback with previously addressed communication theories. Furthermore, the local supervisors should be able to give continuous constructive feedback on communication skills in a real setting follow-up. Teaching and evaluating communication cannot be wholly technical, objective, and numerical, as there exists a significant subjective, individual, and intuitive dimension. For this reason, we also welcome further studies using qualitative methods [33, 44].

We also suggest complementing the approach of the effects of video feedback sessions in this methodology from other viewpoints, such as the preceptors, staff, and real patients [45, 46]. Furthermore, we recommend further research about assessing other skills such as clinical records and time management, in addition to investments in multicentric clinical trials on communication programs and its impact, and further evaluation tools and teaching methodologies for video feedback.

### Limitations

Although the researchers are unsure as to how each variable relates to better effectiveness in the video feedback [2, 20, 47], a limitation of the study was the inability to causally link intervention with any effects. We did not focus on the effectiveness of the methodological variables regarding the effects of the communication program. Moreover, another limitation of this study was the low sample size, leaving us with low power in all analyses. In other words, there is a high likelihood of “false negatives” and the possibility that our results indicate that no difference exists when, in fact, they do. We also had limitations regarding the lack of standardization of raters and SPs, the variations of subjective judgments, and variation in local supervisors.

The assessment instruments used in Portuguese did not go through a rigorous cross-cultural adaptation, but simple translations. Besides, the small sample hampered the ability of the researchers to obtain any validity evidence based on the internal structure of the translated instruments. Therefore, one may infer that a more extended follow-up period would have been necessary to detect a significant improvement in communication attributable to the intervention [43].

## Conclusions

VF taken from real-life settings seems to be associated with a significant increase in self-perceived empathy. It seems that the absence of additional measurable differences may be related to the small sample size and insufficient follow-up time. The main self-reported perceptions by the medical residents suggested that this VF educational intervention has the potential to promote beneficial changes in clinical practice. The mains changes reported: better patient-centered approach, improvement of non-verbal communication, self-confidence, emotional control, behavioral reactions, and better organization of the consultation. Besides, the results suggested that participants may retain such positive changes in their professional practice by incorporating reflective practices.

This study points to some critical elements to consider when preparing a communication program with VF sessions using real consultations. The potential benefits mentioned included the focus in self-perception, identifying learning goals in CCS, and the possibility to look at oneself interacting with a real patient in a real-life setting from a distance, a revisiting point. VF appears to be an opportunity for participants to experience a deeper level of self-assessment, peer-feedback, as well as reflective practices.

Moreover, VF seems to benefit from facilitator that are attentive to the learners’ psychological needs and skilled in relating the feedback with communication theory. Further studies on VF using real-life consultations could make use of inter-institutional collaborations to help circumvent the limitations related to sample size. Given that the complex skills targeted by VF take a long time to develop, future studies on VF would likely benefit from a more extended period of longitudinal follow-up.

## Data Availability

The datasets used and/or analyzed during the current study are available from the corresponding author on reasonable request.

## Abbreviations

ALOBA: Agenda-led outcome-based analysis
CARE: Consultation and Relational Empathy
CCOG: Questionnaire-based in Calgary-Cambridge Observation Guide
CCS: Clinical communication skills
JSPE: Jefferson Scale of Physician Empathy
PBI: Problem-based interviewing
PPC: Perception of Patient-Centeredness
VF: Video feedback

## References

[1] Kurtz S, Silverman J, Draper J (2005). Teaching and learning communication skills in medicine.

[2] Fukkink RG, Trienekens N, Kramer LJC (2011). Video feedback in education and training: putting learning in the picture. Educ Psychol Rev.

[3] Epstein RN (2007). Assessment in medical education. N Engl J Med.

[4] van der Vleuten CPM (1996). The assessment of professional competence: developments, research, and practical implications. Adv Health Sci Educ.

[5] Eeckhout T, Gerits M, Bouquillon D, Schoenmakers B (2016). Video training with peer feedback in real-time consultation: acceptability and feasibility in a general-practice setting. Postgrad Med J.

[6] Asan O, Montague E (2014). Using video-based observation research methods in primary care health encounters to evaluate complex interactions. Inform Prim Care.

[7] Henry SG, Forman JH, Felters MD (2011). How do you know what aunt Martha looks like?’ A video elicitation study exploring tacit clues in doctor–patient interactions. J Eval Clin Pract.

[8] Perron J, Louis-simonet M, Cerutti B (2016). Feedback in formative OSCEs: comparison between direct observation and video-based formats. Med Educ Online.

[9] Hammoud MM, Morgan HK, Edwards ME, Lyon JA, White C (2012). Is video review of patient encounters an effective tool for medical student learning? A review of the literature. Adv Med Educ Pract.

[10] Hergie O, Morrow NC (1986). Using videotape in communication skills training: a critical review of the process of self-viewig. Med Teach.

[11] Gask L (1998). Small group interactive techniques utilizing videofeedback. Int J Psychiatry Med.

[12] Roe P (1980). Training medical students in interviewing skills.

[13] Evans RG, Edwards A, Evans S, Elwyn B, Elwyn G (2007). Assessing the practising physician using patient surveys: a systematic review of instruments and feedback methods. Fam Pract.

[14] Kalish R, Dawiskiba M, Sung YC, Blanco M (2011). Raising medical student awareness of compassionate care through reflection of annotated videotapes of clinical encounters. Educ Health.

[15] Pelgrim EA, Kramer AW, Mokkink HG, van der Vleuten CP (2013). Reflection as a component of formative assessment appears to be instrumental in promoting the use of feedback: an observational study. Med Teach.

[16] Lesser AL (1985). Problem-based interviewing in general practice: a model. Med Educ.

[17] Pendleton D, Schofield T, Tate P, Havelock P (2006). The new consultation -developing doctor-patient communication.

[18] Silverman JD, Kurtz SM, Draper J (1996). The Calgary-Cambridge approach to communication skills teaching. 1. Agenda led outcome-based analysis of the consultation. Educ Gen Pract.

[19] Dowrick P, Dowrick P, Biggs S (1983). Self-modeling. Using video: Psychological and social applications.

[20] Hosford RE, Mills ME, Dowrick PW, Biggs SJ (1983). Video in social skills training. Using video; Psychological and social applications.

[21] Bandura A (1969). Principles of behavior modification.

[22] Carrió FB (2004). Clinical Interview: a practical strategies handbook.

[23] Hattie J, Timperley H (2007). The power of feedback. Rev Educ Res.

[24] Mercer SW, Maxwell M, Heaney D, Watt GC (2004). The consultation and relational empathy (CARE) measure: development and preliminary validation and reliability of an empathy-based consultation process measure. Fam Prac.

[25] Scarpellini GR, Capellato GRF, Da Silva GA (2014). CARE scale of empaty: translation to the Portuguese spoken in Brazil and initial validation results. Med (Ribeirão Preto Online).

[26] Stewart M, Brown JB, Weston WW, McWhinney IR, McWilliam CL, Freeman TR. Patient-centered medicine. Transforming the clinical method. 3rd ed. New York: Radcliffe Publishing; 2014.

[27] Hojat M, Mangione S, Nasca TJ, Cohen MJM, Gonnella JS, Erdmann JB, Veloski JJ, Magee M (2001). The Jefferson scale of physician empathy: development and preliminary psychometric data. Educ Psychol Measur.

[28] Paro HB, Daud-Gallotti RM, Tibério IC, Pinto RM, Martins MA (2012). Brazilian version of the Jefferson scale of empathy: psychometric properties and factor analysis. BMC Med Educ.

[29] Baile WF, Buckman R, Lenzi R, Glober G, Beale EA, Kudelka AP (2000). SPIKES—a six-step protocol for delivering bad news: application to the patient with cancer. Oncologist.

[30] Braun V, Clarke V (2006). Using thematic analysis in psychology. Qualit Res in Psychology.

[31] Cruess SR, Cruess RL (2016). Professionalism as a social construct: the evolution of a concept. J Grad Med Educ.

[32] Hodges BD, Ginsburg S, Cruess R, Cruess S, Delport R, Hafferty F (2011). Assessment of professionalism: recommendations from the Ottawa 2010 conference. Med Teach.

[33] van der Vleuten CP, Schuwirth LW, Scheele F, Driessen EW, Hodges B (2010). The assessment of professional competence: building blocks for theory development. Best Pract Res Clin Obstet Gynaecol.

[34] Rothman AI, Blackmore D, Dauphinee WD (1997). The use of global ratings in OSCE station scores. Adv Health Sci Educ Theory Pract.

[35] Hodges B, Regehr G, Mcnaughton N, Tiberius R, Hanson M (1999). Checklists do not capture increasing levels of expertise. Acad Med.

[36] Pinsky LE, Wipf JE (2000). A picture is worth a thousand words: practical use of videotape in teaching. J Gen Intern Med.

[37] Nilsen S, Baerheim A (2005). Feedback on video recorded consultations in medical teaching: why students loathe and love it – a focus group based qualitative study. BMC Med Educ.

[38] Hulsman RL, van der Vloodt J (2015). Self-evaluation and peer-feedback of medical students’ communication skills using a web-based video annotation system. Exploring content and specificity. Patient Educ Couns.

[39] Norcini JJ (2003). Peer assessment of competence. Med Educ.

[40] Topping KJ (1996). The effectiveness of peer tutoring in further and higher education: a typology and review of the literature. High Educ.

[41] Telio S, Ajjawi R, Regehr G (2015). The “educational alliance” as a framework for reconceptualizing feedback in medical education. Acad Med.

[42] Driessen EW, van Tartwijk J, Govaerts M, Teunissen P, van der Vleuten CP (2012). The use of programmatic assessment in the clinical workplace: a Maastricht case report. Med Teach.

[43] van der Vleuten CPM, Schuwirth LWT, Driessen EW, Govaerts MJB (2014). Twelve tips for programmatic assessment. Med Teach.

[44] Moonen-Van Loon JM, Overeem K, Govaerts MJ, Verhoeven BH, van der Vleuten CP, Driessen EW (2015). The reliability of multisource feedback in competency-based assessment programs: the effects of multiple occasions and assessor groups. Acad Med.

[45] Ten Cate O, Sargeant J (2011). Multisource feedback for residents: how high must the stakes be?. J Grad Med Educ.

[46] Donnon T, Al Ansari A, Al Alawi S, Violato C (2014). The reliability, validity, and feasibility of multisource feedback physician assessment: a systematic review. Acad Med.

[47] Goldie J (2013). Assessment of professionalism: a consolidation of current thinking. Med Teach.

